# The effect of hemispheric lesion location on trunk control

**DOI:** 10.1097/MD.0000000000038589

**Published:** 2024-06-28

**Authors:** Şennur Delibaş Kati, Elif Ayşen Palaz, Yağmur Güneş Gencer, Hanife Hale Hekim, Neriman Temel Aksu, Aylin Yaman, Naciye Füsun Toraman

**Affiliations:** a Department of Neurology, University of Health Sciences, Antalya Health Research Center, Antalya, Türkiye; b Private Artrolife Clinic, Physical Medicine and Rehabilitation, Antalya, Türkiye; c Department of Gerontology, Akdeniz University, Faculty of Health Sciences, Antalya, Türkiye; d Department of Physical Medicine and Rehabilitation, University of Health Sciences, Antalya Health Research Center, Antalya, Türkiye; e Department of Physiotherapy and Rehabilitation, Faculty of Health Sciences, Akdeniz University, Antalya, Türkiye.

**Keywords:** hemispheric localization, lateropulsion, stroke, trunk control

## Abstract

**Background:**

Trunk control is the basic component of postural control, and achieving trunk control is a complex process that can be achieved by dynamically building and maintaining neuromuscular function. Lateropulsion, which is also defined as the body falling to one side, is considered an important condition that is frequently encountered after stroke and affects trunk control. It is known that there are differences in the regulation of postural control and trunk control according to hemispheric localization. We had a very specific group of patients and tried to find out the outcomes prospectively in this study.

**Methods:**

The patients were divided into 2 groups those with right hemisphere lesions (Group 1) and those with left hemisphere lesions (Group 2). Comorbidity and cognitive function were evaluated using the Charlson Comorbidity Index (CMI) and Standardized Mini-Mental State Test (SMMSE). Activities of daily living were evaluated using the Turkish version of the Modified Barthel Index (MBI). The Stroke Rehabilitation Assessment of Movement Instrument (STREAM) test was used to assess trunk control and the Brunnstrom (BS) test was used to assess motor functions.

**Results:**

There was a significant difference between Groups 1 and 2 in terms of STREAM in lower extremity scores were higher in Group 2 (*P* < .05). The number of patients in BS lower extremity Stages IV-VI was higher in Group 1 and Group 2 (*P* < .05). It was determined that upper extremity, lower extremity and Total STREAM scores and BS Hand stage in Group 2 were significantly higher than Group 1 in patients with total middle cerebral artery (MCA) affected(*P* < .05).

**Conclusion:**

It was determined that trunk control was more affected in patients with right hemispheric lesions. Additionally, trunk control is significantly affected in patients with total MCA lesions.

## 1. Introduction

Trunk control is the basic component of postural control, and achieving trunk control is a complex process that can be ensured by dynamically building and maintaining neuromuscular function. poststroke trunk control is affected by different levels and features according to the affected hemisphere. Lateropulsion -meaning “lateralis” (side) and “pulsus” (to push)in Latin.-, which is also defined as the body falling to one side, is considered an important condition that is frequently encountered after stroke and affects trunk control.^[1]^ There are differences in the regulation of postural control and trunk control according to hemispheric localization.^[1]^ It has been reported that postural control is worse in patients with right hemisphere lesions.^[2–5]^ While postural instability in the trunk is more dominant in patients with right hemisphere lesions, trunk apraxia is more prominent in patients with left hemisphere lesions.^[6,7]^ It has also been demonstrated that there is an increase in lateral instability in patients with right temporoparietal junction lesions and, accordingly, the ability to stabilize and maintain the trunk in the frontal plane decreases.^[6]^ All this evidence suggests that right hemisphere lesions have a dominant role in trunk control.^[7]^

Although there are studies investigating the relationship between trunk control and the affected hemisphere after stroke, the number of studies evaluating the relationship between postural and trunk control according to intrahemispheric lesion location is limited.^[8,9]^ Anterior structures such as the frontal area, thalamus, and parietal area, and posterior structures such as the brain stem and cerebellum have separate functions in trunk control.^[10]^ Despite studies evaluating the effect of intrahemispheric lesion location on trunk control, they are few in number and are not of a prospective nature. In addition, studies in which possible clinical conditions (aphasia, apraxia, visual and auditory disorders, bilateral hemiplegia, musculoskeletal system disorders, etc) that may affect the evaluation of trunk control are isolated and excluded that may mask the evaluation of the effect of the intrahemispheric lesion on trunk control or lead to false evaluations were not followed in literature.

In the present study, we created a patient group in which visual, auditory, mental, or physical conditions were accompanied along with poststroke hemiplegia which might affect the assessment of trunk control. Areas thought to play a role in trunk control in this patient group were classified according to vascular involvement. We aimed to evaluate the effect of lesions in these areas on trunk control according to intrahemispheric localization.

## 2. Materials and methods

**Figure 1.**
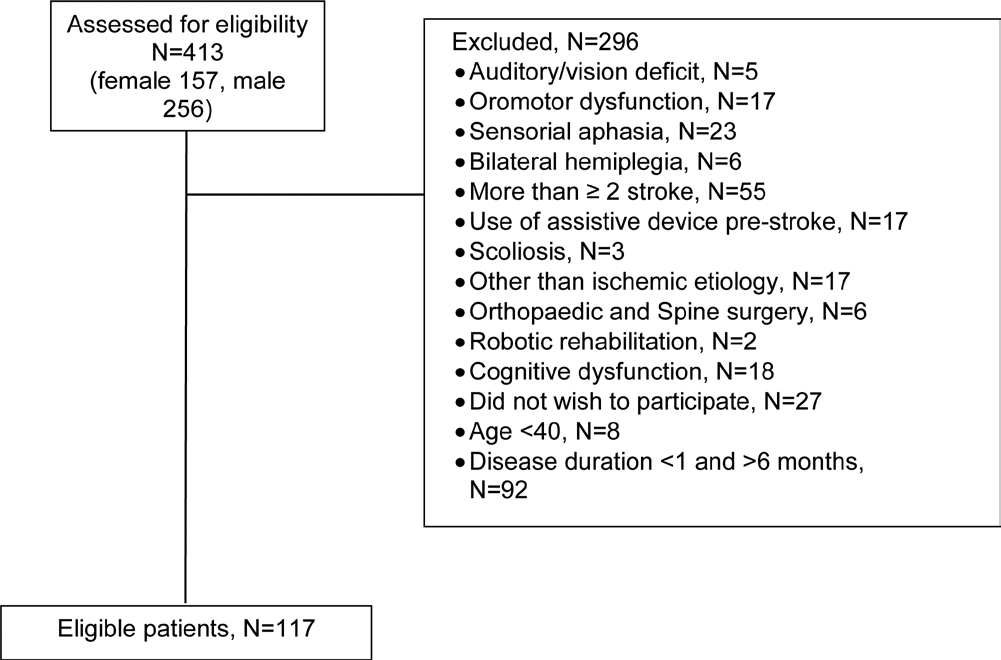
Study flowchart.

This prospective study was conducted at the Physical Medicine Rehabilitation and Neurology outpatient clinics of XXX between August 2018 and January 2020. Initially, a total of 413 stroke patients were screened. Conditions that may affect the clinical evaluation of trunk control were specified to optimally measure the effect of the localization of the hemispheric lesions targeted on trunk control. These included sensory aphasia that may affect the evaluation due to speech and comprehension disorders, apraxia, visual and auditory disorders, the presence of bilateral hemiplegia in which trunk control cannot be fully achieved, having more than one stroke, having undergone robotic surgery that may affect the evaluation due to clinical improvement after stroke, having musculoskeletal system (i.e., previous orthopedic surgery, limb inequality, deformity) disorders that may affect trunk-extremity control other than cerebral causes, the presence of dementia findings and immobilization for a pre-stroke reason. Patients with at least one of these conditions were excluded from the study. Those younger than 40 years of age and those who were in the poststroke evaluation for less than 1 month or more than 6 months were also excluded from the study. Finally, a total of 117 patients who met the inclusion criteria were included on a voluntary basis (Fig. 1). A written informed consent was obtained from each patient. The study protocol was approved by the XXX Ethics Committee (2018-157). The study was conducted by the principles of the Declaration of Helsinki.

The patients were divided into 2 groups as those with right hemisphere lesions (Group 1) and those with left hemisphere lesions (Group 2). Then, areas in each hemisphere that may affect trunk control were identified and lesions in these areas were classified into 6 localizations according to the affected vascular area: total middle cerebral artery (MCA) area, frontal area with cortical MCA area, lenticulostriate artery area, thalamic area, brain stem area, and occipitocerebellar area.

Demographic data of the patients such as age, sex, body mass index (BMI), education status, marital status, smoking and alcohol use, dominant extremity, presence and region of spasticity, limitation in joint range of motion (ROM), presence of pain(pain was assessed by questioning whether present or not), poststroke disease duration, and rehabilitation period were recorded. Comorbidity was evaluated using the Charlson Comorbidity Index (CMI) cognitive function was evaluated using the Standardized Mini-Mental State test (SMMSE), and those who scored > 24 were considered cognitive normal.^[11–13]^ Activities of daily living were evaluated using the Turkish version of the Modified Barthel Index (MBI), which consists of 10 items and some of these items specially evaluate daily living activities such as walking, climbing stairs, dressing, eating, bathing, and toilet use.^[14]^

The Stroke Rehabilitation Assessment of Movement Instrument (STREAM) test consisting of 30 items was used in the trunk control evaluation and the Brunnstrom (BS) test was used in the motor function evaluation.^[15,16]^

### 2.1. Statistical analysis

Statistical analysis was performed using the SPSS software version 25.0 software (IBM Corp., Armonk, NY, USA). Descriptive data were presented in mean ± standard deviation (SD), median (min-max) or number and frequency. Normality assumption was checked by examining histogram, q-q plot, skewness, and kurtosis values with the Kolmogorov-Smirnov test. In the analysis of the difference between the numerical data of the 2 groups, the Independent samples *t*-test was used, when the data conformed to the normal distribution, while the Mann–Whitney U test was used for non-normally distributed variables. For categorical data, the Pearson chi-square was used when the ratio of cells with an expected value <25%, and Fisher exact test was used when it was higher than 20%. The Yates continuity correction was used, when the expected value was <25. The Kruskal-Wallis H test was used for intragroup analysis, and the Bonferroni-Dunn procedure was performed for pairwise comparisons for significant results. The Mann–Whitney *U* test was carried out for the comparison of subgroup analysis. *P* values of < .05 and < .008 were considered statistically significant for inter-group comparisons and intragroup analysis, respectively.

## 3. Results

There were 26 females and 40 males in Group 1 and 23 females and 28 males in Group 2. There was no significant difference between the groups in terms of age, sex, BMI, education status, marital status, smoking and alcohol use, extremity dominance, presence of spasticity, joint ROM, disease duration, rehabilitation period, ambulation status, CMI, SMMSSE, and MBI scores (*P* > .05) However, the number of patients having pain was significantly higher in Group 1 than Group 2 (*P* < .05) (Table 1).

**Table 1:** Baseline evaluation of the groups.

		Group I (Right) N = 66 N (%) median (min–max)	Group II(Left) N = 51 N (%) median (min–max)	*P*
Age, year		64.9 ± 10.3	64.7 ± 11.4	e.952
Gender, F/M		26 (39)/40 (61)	23 (45)/28 (55)	a.535
BMI, kg/m^2^		26.8 (18.4–35)	26.3 (21.6–36.9)	f.642
Educational status	Illiterate	9 (14)	9 (18)	c.486
	Primary school	37 (56)	30 (59)	
	High school	14 (21)	7 (14)	
	University	6 (9)	5 (10)	
Marital status	Married	50 (76)	38 (75)	d1.000
	Single	16 (24)	13 (26)	
Smoking		32 (49)	19 (37)	b.168
Alcohol use		9 (14)	12 (24)	b.168
Dominant extremity. right/left		59 (89)/7 (11)	46 (90)/5 (10)	b.887
Spasticity presence		24 (36)	18 (35)	d1.000
ROM limitation presence		21 (32)	10 (20)	d.203
Pain presence		27 (41)	11 (22)	d **.044**
Disease duration, month		2 (1–6)	2 (1–6)	f.447
Rehabilitation duration, session		26 (10–72)	28 (13–90)	f.410
Ambulatuar status	Ambulatuar	29 (44)	26 (51)	d.305
	With assist	18 (27)	16 (31)	
	Non-ambulatuar	19 (29)	9 (18)	
CMI, score		3 (1–5)	3 (1–5)	f.958
SMMSE, score		28 (24–30)	27 (24–30)	f.248
MBI, score		78.5 (7–100)	78 (30–100)	a.182

BMI = body mass index, CMİ = charlson, comorbidity index, SMMSEF = standardized mini-mental state examination female, M = Male, ROM = range of motion.

a Pearson chi-square test,

b Likelihood ratio,

c linear by linear association,

d Yates continuity correction,

e independent-*t* test,

f Mann–Whitney *U* test.

Scores of daily living activities, trunk control, and motor function are summarized in Table 2 accordingly. STREAM, one of the scoring systems used, gives us information about our lower extremity, upper extremity, and total evaluation. BS stage gives us the chance to evaluate hand function. There was no significant difference between Groups 1 and 2 in terms of STREAM upper extremity, basic mobility, and total scores (*P* > .05); however, lower extremity scores were higher in Group 2 (*P* < .05). There was no significant difference in the BS upper extremity and hand stages (*P* > .05); however number of patients in BS lower extremity stages IV–VI was higher in Group 1 and 2 (*P* < .05) (Table 2).

**Table 2:** Daily living activities, trunk control and motor function of the groups.

		Group I, N = 66 median (min–max) N [%]	Group II, N = 51 median (min–max) N [%]	*P*
STREAM, score	UE	17.5 (0–20)	20 (0–20)	a.071
	LE	**13.5 (0–20**)	**17 (0–20**)	a **.028**
	BM	24 (2–30)	26 (0–30)	a.162
	Total	50 (3–70)	61 (0–70)	a.060
BS, UE, stage	I–III	29 [44]	14 [28]	b.101
	IV–VI	37 [56]	37 [73]	
BS, hand, stage	I–III	30 [46]	20 [39]	b.626
	IV–VI	36 [55]	31 [61]	
BS, LE, stage	I–III	**19 [29]**	**6 [12]**	c **.045**
	IV–VI	**47 [71]**	**45 [88]**	

BM = basic mobility, BS = Brunnstrom, LE = = lower extremity, MBI = modified barthel index, STREAM = stroke rehabilitation assessment of movement instrument, UE = upper extremity.

a Mann–Whitney *U* test,

b Yates continuity correction,

c Likelihood ratio.

According to the regional distribution of right hemisphere lesions, 36% of the lesions were located in the total MCA area, 18% in the frontal with cortical MCA area, 11% in the lenticulostriate artery area, 12% in the thalamic area, 20% in the brain stem area, and 3% in the occipitocerebellar area. According to the regional distribution of left hemisphere lesions, 37% were located in the total MCA area, 4% in the frontal with cortical MCA area, 14% in the lenticulostriate artery area, 14% in the thalamic area, 23% in the brain stem area, and 8% in the occipitocerebellar area (Table 3). When STREAM and BS stages of all these 6 regions were compared separately according to right or left hemisphere lesions, it was found statistically significant that patients with thalamic infarction in Group 1 had better BS upper extremity stages (*P* < .008). Apart from this, no significant difference was found in the group analyses according to the location of the intrahemispheric lesion. (*P* > .008). When the affected regions in the hemispheric areas were compared according to Group 1 and Group 2, it was determined that upper extremity, lower extremity and Total STREAM scores and BS Hand stage in Group 2 were significantly higher than Group 1 in patients with total MCA affected(*P* < .05).

**Table 3:** Group differences in relation to STREAM and Brunnstom according to the vascular lesion.

		TMCA		FCMCA		LSA		T		BS		OC		
		Group I N = 24	Group II N = 19	Group I N = 12	Group II N = 2	Group I N = 7	Group II N = 7	Group I N = 8	Group II N = 7	Group I N = 13	Group II N = 12	Group I N = 2	Group II N = 4	
STREAM, score	UE	4.5 [0–20]	17 [0–20]	18.5 [0–20]	20 [20–20]	6 [0–20]	18 [0–20]	20 [0–20]	16 [15–20]	20 [1–20]	20 [4–20]	20 [20–20]	20 [8–20]	‖p_1_ = 0.009 ‖p_2_ = 0.264
		* ***P* = .029**		*P = .352		*P = .535		*P = .232		*P = .979		*P = 1.000		
	LE	12 [0–20]	18 [0–20]	13 [0–20]	20 [20–20]	9 [4–20]	17 [0–19]	19 [0–20]	16 [9–20]	17 [3–20]	16 [12–20]	19 [18–20]	18 [4–20]	‖p_1_ = 0.136 ‖p_2_ = 0.253
		* ***P* = .030**		*P = .132		*P = .223		*P = .626		*P = .868		*P = .784		
	BM	18.5 [3–30]	27 [0–30]	27.5 [4–30]	30 [30–30]	17 [2–29]	24 [4–30]	28.5 [3–30]	26 [15–30]	25 [5–30]	25 [18–30]	27 [24–30]	22.5 [8–30]	‖p_1_ = 0.351 ‖p_2_ = 0.399
		*P = .104		*P = .132		*P = .442		*P = .559		*P = .978		*P = 1.000		
	Total	36.5 [4–70]	62 [0–70]	55 [4–70]	70 [70–70]	30 [14–69]	55 [4–67]	66 [3–70]	58 [40–70]	61 [9–69]	56 [48–70]	66 [62–70]	60.5 [20–70]	‖p_1_ = 0.101 ‖p_2_ = 0.184
		* ***P* = .045**		*P = .088		*P = .370		*P = .447		*P = .785		*P = .639		
BS, UE, stage	I–III	17 (71)	8 (42)	4 (33)	1 (50)	3 (43)	2 (29)	1 (13)	1 (14)	4 (31)	2 (17)	–	–	‡p_1_ = 0.012 ‡p_2_ = 0.271
	IV–VI	7 (29)	11 (58)	8 (67)	1 (50)	4 (57)	5 (71)	7 (88)	6 (86)	9 (69)	10 (83)	2 (100)	4 (100)	
		†*P* = .058		§*P* = .604		§*P* = .500		§*P* = .733		§*P* = .363		–		
BS, hand, stage	I–III	18 (75)	7 (37)	4 (33)	2 (100)	3 (43)	4 (57)	**1 (13**)	3 (43)	4 (31)	3 (25)	–	1 (25)	‡**p**_**1**_** = 0.004** ‡p_2_ = 0.113
	IV–VI	6 (25)	12 (63)	8 (67)	–	4 (57)	3 (43)	**7 (88**)	4 (57)	9 (69)	9 (75)	2 (100)	3 (75)	
		† ***P* = .012**		§*P* = .165		§*P* = .500		§*P* = .231		§*P* = .613		–		
BS, LE, stage	I–III	11 (46)	4 (21)	2 (17)	–	3 (43)	1 (14)	1 (13)	1 (14)	2 (15)	–	–	–	‡p_1_ = 0.128 ‡p_2_ = 0.316
	IV–VI	13 (54)	15 (79)	10 (83)	2 (100)	4 (57)	6 (86)	7 (88)	6 (86)	11 (85)	12 (100)	2 (100)	4 (100)	
		§*P* = .116		§*P* = .725		§*P* = .280		§*P* = .733		§*P* = .260		–		

Numerical data are given as median [min–max] and categorical data as numbers (%).

BM = basic mobility, BS = Brunnstrom, BS = brain stem area, FCMCA = frontal with cortical middle cerebral artery, LE = lower extremity, LSA = lenticulostriate artery, OC = occipitocereballar area, STREAM = stroke rehabilitation assessment of movement instrument, T = thalamic, TMCA = total middle cerebral artery, UE = upper extremity.

a Pearson chi-square test,

* Mann–Whitney *U* test,

† chi-square test,

§ Fisher test,

‡ chi-square Yatex continuity correction,

‖ Kruskal-Wallis, *P* = group difference in relation to area, *P* < .05, p_1_ = within right hemisphere in relation to area, *P* < .008 p_2_ = within left hemisphere in relation to area, *P* < .008.

## 4. Discussion

Trunk control is affected by the lesions in the brain. In our study which is stroke-based, right hemispheric lesions have a significantly negative effect on trunk control. We came to this conclusion in the light of the following 3 statistical data. These are the presence of pain more on the right side, STREAM lower extremity assessment was found to be low in patients with right hemispheric involvement, and It has also been shown that right hemispheric involvement is worse upper extremity, lower extremity and Total STREAM scores and BS Hand stage in patients with total MCA. Apart from these 3 findings, it was statistically proven that BS hand stages were better in the group with right hemispheric thalamic involvement. These are the main results of our study.

Lateropulsion is the term used to describe actively pushing the body along the midline toward the more affected side and/or actively resisting weight shift to the less affected side.^[17]^ Following a unilateral stroke, the patient’s trunk balance becomes deteriorates, particularly toward the contralateral side of the lesion.^[8]^ It has been emphasized that postural balance is impaired due to the fact that vestibular functions are affected more in cases with right hemisphere lesions.^[2]^ The higher incidence of lateropulsion in cases with posterior insula, operculum, and superior temporal gyrus involvement has been attributed to the fact that right hemisphere lesions are more associated with vestibular function.^[2]^ There is truncal apraxia and related posture disorder in the presence of apraxia in left hemisphere lesions, and postural instability with more complex sensory involvement in right hemisphere lesions.^[7]^ In our study, STREAM lower extremity evaluation results of patients with right hemispheric involvement were found to be significantly lower than those of patients with left hemispheric involvement. This supports the finding that right hemispheric involvement affects the lower extremities more and especially causes lateropulsion. In addition, according to BS lower extremity stages, complex movement combinations or near-normal muscle evaluation results which mean good scores, are statistically significant higher in patients in both group.

In a retrospective study investigating the effects of stroke lesion location and size on lateropulsion, also the size of right hemisphere lesions was found to be an important factor that was strongly related to lateropulsion.^[18]^ In addition, there is a significant balance impairment in balance tests in patients with right hemisphere lesions. Therefore, it is necessary to plan rehabilitation strategies to correct vertical posture in stroke patients and to understand the pathophysiology of lateropulsion. In rehabilitation practice for balance and posture, patients with right hemisphere lesions recover more slowly and later than those with left hemisphere lesions. Those with right hemisphere lesions, particularly those with neurological involvement of pain, cannot achieve as a good treatment response as those with left hemisphere lesions.^[19]^ In the present study, the size of the lesions was not evaluated, but the presence of pain and its association with low treatment response was not observed in patients with right hemispheric involvement in the literature. In this case, the presence of pain may not be considered a valid prognostic factor for low treatment response in patients with right hemispheric involvement.

When assessed according to vascular lesion or in other words intrahemispheric localization, the relationship between the area of stroke involvement and trunk control is not clear. For example, the insular cortex has been thought to be implicated in trunk control. It is a region associated with spatial perception and spatial orientation.^[20]^ However, it is not directly related to lateropulsion in isolated lesions of the insular cortex.^[20]^ This can be explained by the fact that different anatomical structures affect lateropulsion or that these structures have complex relationships. It has been shown that the inferoparietal lobe constitutes an important localization in the comparison of patients with and without signs of lateropulsion. In other words, inferoparietal lobe lesion involvement is seen as an important determinant in the development of lateropulsion.^[21]^ Both the insular cortex and the inferoparietal area are the areas affected in MCA lesions. In our study, we observed that the scores for upper extremity, lowerextremity and total STREAM, as well as the BS Hand stage, were notably lower in patients within group 1, particularly those with total MCA involvement. This finding can be considered the most significant data from our study. Consequently, total MCA involvement emerges as a crucial factor contributing to lower functional scores, impaired trunk control, and potentially lateropulsion. In other words, our research demonstrates that localization, as well as lateralization, has a significant impact on trunk control. We did not find any similar studies in the existing literature.

Thalamus lesions are one of the frequently affected areas in stroke patients. The thalamus, which acts as a gate for all afferent messages, also processes vestibular inputs and provides the interrelationship of different areas of the brain. In lesions in which the thalamic area is affected, high-grade sensory defects such as spatial neglect, lateropulsion, or thalamic astasia can be observed.^[22]^ In our study, the rate of thalamus involvement was low (Group 1 12%, Group 2 14%). Therefore, we believe that the effect of thalamic lesions on lateropulsion and trunk control should be evaluated in a larger sample. However, when patients with thalamic involvement were evaluated, it was determined that functional scores were higher, especially in patients with right hemispheric involvement.^[22]^ In our study, similar to the literature, we found that the BS hand stages of patients with thalamic region involvement were significantly higher.

The vestibular cortex, which is different from other sensory cortices, consists of a network of several different areas.^[18]^ Its core region, the parietoinsular vestibular cortex, is located in the posterior insula and retroinsular region and includes the parietal operculum.^[19]^ The entire network consists of vestibular, visual, and somatosensory senses and this cortical area has 2 major functions; spatial orientation and self-motion perception. The peripheral and central vestibular systems are organized bilaterally. Structural and functional vestibular dominance refers to the right hemisphere in right-handers and the left hemisphere in left-handers. This result explains why right hemispheric lesions often lead to hemispatial neglect and lateropulsion in right-handers. Although the vestibular cortex is represented in both hemispheres, there is only one general perception of body position and movement. However, it is not possible to make a clear anatomical distinction of vestibular function.^[23]^ In our study, we observed that right hemispheric involvement was predominant in most of the statistically significant results (patients with more pain, patients with lower STREAM lower extremity scores, and patients with lower BS lower extremity stages). This supports a possible relationship between right hemispheric involvement and the involvement of the vestibular cortex mentioned above, but on the other hand, we had more results with no difference between both hemispheric involvement. Therefore, the relationship between right hemispheric involvement and the vestibular cortex is still an area in need of clarification and scientific study.

The main strength of this study is the consideration of factors that may affect trunk control for patient inclusion and exclusion criteria and evaluating the relationship between intrahemispheric involvement and trunk control. One of the limitations is, however, the relatively small sample size. Although our center is a regional stroke center, we could not obtain the number of subgroups that would enable us to analyze the study at the intrahemispheric level. Another limitation is that some criteria were used to exclude the majority of stroke patients and it is not easy to provide this isolation in daily practice. In addition, the lack of information about the rehabilitation protocol was considered as another limitation. Nevertheless, we believe that our study provides an additional contribution to the body of knowledge in the literature on this subject.

In conclusion, it is known that trunk control is more impaired in right hemispheric involvement and we think that the low lower extremity function of patients with right hemispheric involvement in our study may be the main reason for this. In addition, It has been shown that trunk control is affected in patients whose total MCA area is affected in right hemispheric involvement, and this is a finding that we think is important for more specific studies in the future.

## Author contributions

**Conceptualization:** Şennur Delibaş Kati, Neriman Temel Aksu, Aylin Yaman, Naciye Füsun Toraman.

**Data curation:** Şennur Delibaş Kati, Elif Ayşen Palaz, Hanife Hale Hekim.

**Formal analysis:** Şennur Delibaş Kati, Yağmur Güneş Gencer, Neriman Temel Aksu, Naciye Füsun Toraman.

**Funding acquisition:** Şennur Delibaş Kati, Elif Ayşen Palaz, Naciye Füsun Toraman.

**Investigation:** Şennur Delibaş Kati, Hanife Hale Hekim, Neriman Temel Aksu, Aylin Yaman, Naciye Füsun Toraman.

**Methodology:** Şennur Delibaş Kati, Hanife Hale Hekim, Aylin Yaman, Naciye Füsun Toraman.

**Project administration:** Şennur Delibaş Kati.

**Resources:** Şennur Delibaş Kati, Yağmur Güneş Gencer, Hanife Hale Hekim.

**Software:** Şennur Delibaş Kati, Yağmur Güneş Gencer, Hanife Hale Hekim, Neriman Temel Aksu.

**Supervision:** Şennur Delibaş Kati, Hanife Hale Hekim, Aylin Yaman, Naciye Füsun Toraman.

**Validation:** Şennur Delibaş Kati, Yağmur Güneş Gencer, Aylin Yaman, Naciye Füsun Toraman.

**Visualization:** Şennur Delibaş Kati, Elif Ayşen Palaz, Yağmur Güneş Gencer, Neriman Temel Aksu.

**Writing – original draft:** Şennur Delibaş Kati, Hanife Hale Hekim, Naciye Füsun Toraman.

**Writing – review & editing:** Şennur Delibaş Kati, Hanife Hale Hekim, Naciye Füsun Toraman.
